# La Charité

**Published:** 1830-10-01

**Authors:** 


### LA CHARITE.

#### I. Excision of a Carious Rib.*

*Case.* Louis Evrard, shoemaker, aged 38, was admitted into La Charité on the 23d of March, with a fistulous opening on the right side of the chest, leading down to the fifth rib. The latter, when examined by the probe, felt rough, denuded, and carious. A considerable quantity of puriform matter was discharged from the fistulous opening; the patient was debilitated, thin, and suffered from a troublesome cough, with expectoration of thick mucous sputa; but no positive sign of phthisis pulmonalis was present.

On the 24th of April M. Roux pro-

* Journ. Hebdomadaire, No. 86.

ceeded to remove the rib. All the soft parts covering it were included between two semi-elliptical incisions, extending from the border of the axilla to near the sternum, and passing immediately under the mamma. By these incisions and the removal of the integuments included between them, the rib was exposed for the extent of five inches; the limits of the carious portion ascertained, and the latter, four inches in length, cut out by means of the chain saw; another small portion near the sternum presenting a suspicious appearance was also taken away. The pleura costalis adhered as usual to the inferior border of the bone, but above it was thrust inwards by a moderate collection of pus, which had no communication with the pleural cavity. The rib was quite carious, the superior border in particular and the internal surface being rough, deprived of its superficial laminæ, and chiefly affected about midway between its two extremities. The wound was simply dressed, and for two or three days the patient appeared to be doing well. Then, however, dyspnœa and symptoms of pleuritis on the right side appeared, and death speedily supervened.

*Sectio Cadaveris*, The right side of the chest contained a considerable quantity of sero-purulent fluid with some flakes of recent lymph. The fluid was confined to the two lower thirds of the pleural cavity, the upper being closed from old and firm adhesions. The upper part of the lung contained many large and half-softened tubercles, principally situated opposite the second, third, and fourth ribs, which were all carious and broke with the greatest facility. The pleura at this part still continued sound. The apex of both lungs was loaded with tubercles, chiefly of the granular kind; some of them were softened, others were not; there was nothing like a vomica. No disease was discoverable in other organs.

The reporter remarks that he is not aware of the operation having been performed more than once in France, which was by Richerand in 1818. The patient died. Joshua Aymar, a surgeon of Grenoble, twice excised several carious ribs with success. The first patient was a woman, forty years of age, in whom the eighth and ninth ribs were diseased; the second, a captain, whose fifth, sixth, and seventh were affected. M. Cittadini has more recently published five cases of successful excision of one or more ribs. In all he opened the cavity of the pleura, and one patient nearly died from the admission of air into it. In the present case of M. Roux's, the tubercles of the lungs must undoubtedly be considered as contributing essentially to the unfavourable character of the case, and the fatal pleuritis. At the same time, the result is calculated to point out the uncertainty that must always hang over this operation, and deter practitioners from engaging in it wantonly or without the most cautious deliberation. We have witnessed one unsuccessful attempt at excision of part of a carious rib, complicated, as it turned out, with an opening into the chest. The patient died, but whether from the immediate effects of the operation we cannot positively say.

### II. Removal of great part of the Lower Jaw—Improvements on the common Operation.

*Case.* A country woman, ætatis 27, was admitted into La Charité, on the 1st of May, with a knobbed, irregular tumour of the gum and body of the lower jaw on the left side. It was hard in some parts, soft in others, and appeared to embrace both sides of the jaw from the symphysis of the chin to the last molar tooth. The mouth was distorted, the tongue pushed over to the opposite side, deglutition difficult, and the articulation of some words impaired. It had commenced with violent pain in the teeth of the left side, which continued for several years without any permanent amendment. The maxillary tumour had been coming on for two years prior to her admission. On the 8th of May, M. Roux proceeded to remove the diseased portion of the jaw. He began the operation by thrusting the point of a straight bistoury through the cheek, about half an inch below the edge of the lower lip, prolonging the incision as far as the region of the os hyoides, and carrying it from thence in a curved direction to just below the projection of the left malar bone. A crescentic flap was thus formed, the convexity looking downwards, and the two extremities extending from the chin to the prominence of the cheek. The flap was detached from the subjacent parts by a rapid dissection from below upwards, by which the maxillary bone and the tumour were perfectly exposed. A common saw was then made to work upon the jaw between the right canine tooth and contiguous incisor, and with some little difficulty the bone was cut across. The other division was made a little before the last molar tooth, and beyond the limits of the disease, which was done with the chain saw. The diseased parts were soon dissected out, several small vessels secured, and the integuments brought together by seven separate sutures. All has hitherto done well, and the wound is now nearly healed.

On examining the tumour, it was found to consist of a fibrous mass, alternately reddish and white, enveloping all the portion of bone that had been removed. This had acquired a spongy texture and considerable size, resembling the medullary sarcoma, and communicating at intervals with the dental alveoli, the whole being encased in a thin envelope of firm consistence. The characteristic appearances were particularly well marked at the extremity of the excised bone corresponding to what remained attached to the ramus of the jaw, a circumstance calculated to inspire well founded dreads of the incomplete eradication of the disease. The reporter is of opinion that the disease began in the internal periosteum of the maxilla, and is fungoid and malignant in its nature.

The reporter remarks that M. Roux intends removing the remaining diseased part of the bone at a future opportunity. He thinks that an incision directed obliquely on the ramus will enable the chain saw to work easily and effectually. The reporter also eulogises the idea of not dividing the angle of the lips as is usually done, but beginning the incision below it. The chain saw is not forgotten in this gentleman's laudatory observations. If the disease was really fungus hæmatodes, we would not give much for the patient's chances of a cure.

### III. DISEASE OF THE TESTICLE. CASTRATION.

We are disposed to notice this case, to shew that the French surgeons are yet far behind us, in the diagnosis and treatment of diseases of the testicle, and we believe of malignant tumours in general.

*Case.* Jacques Byard, ætatis 28, a carpenter, was admitted into La Charité on the 21st of December, 1829, with considerable enlargement of one of the testicles. It was oval in shape; very heavy; about four inches it its long diameter, from above downwards; the skin was tense, shining, unadherent; the cord was unaffected. The disease had been coming on eight months, and was attributed by the patient to a blow. This had been followed by violent pain which disappeared spontaneously, but was succeeded in the course of a week by a little towards the centre of the testicle, and continued to advance. For a month the patient had suffered from lancinating pains, "the almost pathognomonic symptom of cancerous affections."

Now, although the case was to all appearance a very unfavourable one, most well-informed English surgeons would have given the patient the chance, which a moderate mercurial course affords. We may in general, by carefully considering the symptoms and accurately sifting the history, form a tolerably correct opinion on the nature of most cases of diseased testicle. But still a degree of uncertainty must in the majority of instances impend over the diagnosis, and the most experienced surgeons are every now and then deceived. Unless then the malignant character of the affection be indisputable, we hold that it is generally better to submit the individual to a gentle course of mercury, conjoined with sarsaparilla or tonics if the health be bad. By this proceeding we shall seldom do harm, and occasionally we save a testis which would otherwise have been removed. This we know to be the practice and opinion of the best surgeons in this country.

However, these considerations had no weight on M. Roux, and on the 26th of December he performed castration. Two ligatures were placed upon the cord, which was cut across by means of scissors—the wound was dressed simply —and the patient did well. Our readers will see that in their mode of performing this operation the French are yet behind us. The vessels of the cord are always tied separately here, and M. Roux's mode of cutting the cord with scissors, is surely awkward looking, and if we may venture to coin a word, unsurgical. On making a section of the amputated testicle, it was found to consist chiefly of a dense, compact structure, of lardaceous consistence, but reddish and fibrous in appearance. It seemed as if the gland of the testis had become hypertrophied, without preserving everywhere its natural texture. In the centre were scattered some whitish, tubercular-looking masses, of different dimensions. The reporter would be inclined to say that the white matter was of medullary character, but he does not positively affirm it. These statements strengthen our criticisms on the practice of proceeding immediately to the operation. We do not pretend that mercury would have cured this disease; we think it would not. But still we cannot before operation see into a testicle, and where there is a chance, it is, cæteris paribus, right to give it.

